# A Nomogram for Predicting Acute Respiratory Failure After Cervical Traumatic Spinal Cord Injury Based on Admission Clinical Findings

**DOI:** 10.1007/s12028-021-01302-4

**Published:** 2021-08-03

**Authors:** Yongfan Xie, Yongyi Wang, Yong Zhou, Mingxing Liu, Shengli Li, Yue Bao, Wenbo Jiang, Siwei Tang, Fangbao Li, Hao Xue, Luo Li, Xingyuan Gong, Yongliang Liu, Weimin Wang, Tong Li

**Affiliations:** 1 Department of Neurosurgery, Qingdao Municipal Hospital (Headquarters), No. 1 Jiaozhou Road, Qingdao, 266011 Shandong People’s Republic of China; 2 School of Medicine, Qingdao University, No. 308 Ningxia Road, Qingdao, 266011 Shandong People’s Republic of China; 3 Department of Neurosurgery, Qingdao Municipal Hospital, No. 5 Donghai Zhong Road, Qingdao, 266071 Shandong People’s Republic of China; 4 Department of Neurosurgery, Neuro Intensive Care Unit, Qingdao Municipal Hospital, No. 5 Donghai Zhong Road, Qingdao, 266071 Shandong People’s Republic of China; 5 Department of Neurosurgery, Qilu Hospital of Shandong University and Institute of Brain and Brain-Inspired Science, Shandong University, No.107 Wenhua Xi Road, Jinan, Shandong People’s Republic of China; 6 Department of Neurosurgery, Binzhou Medical University Hospital, Binzhou, 256603 Shandong People’s Republic of China

**Keywords:** Cervical traumatic spinal cord injury, Acute respiratory failure, Inflammation, Malnutrition, Nomogram

## Abstract

**Objectives:**

Acute respiratory failure (ARF) is a common medical complication in patients with cervical traumatic spinal cord injury (TSCI). To identify independent predictors for ARF onset in patients who underwent cervical TSCI without premorbid respiratory diseases and to apply appropriate medical supports based on accurate prediction, a nomogram relating admission clinical information was developed for predicting ARF during acute care period.

**Methods:**

We retrospectively reviewed clinical profiles of patients who suffered cervical TSCI and were emergently admitted to Qingdao Municipal Hospital from 2014 to 2020 as the training cohort. Univariate analysis was performed using admission clinical variables to estimate associated factors and a nomogram for predicting ARF occurrence was generated based on the independent predictors from multivariate logistic regression analysis. This nomogram was assessed by concordance index for discrimination and calibration curve with internal-validated bootstrap strategy. Receiver operating characteristic curve was conducted to compare the predictive accuracy between the nomogram and the traditional gold standard, which combines neuroimaging and neurological measurements by using area under the receiver operating characteristic curve (AUC). An additional 56-patient cohort from another medical center was retrospectively reviewed as the test cohort for external validation of the nomogram.

**Results:**

162 patients were eligible for this study and were included in the training cohort, among which 25 individuals developed ARF and were recorded to endure more complications. Despite the aggressive treatments and prolonged intensive care unit cares, 14 patients insulted with ARF died. Injury level, American Spinal Injury Association Impairment Scale (AIS) grade, admission hemoglobin (Hb), platelet to lymphocyte ratio, and neutrophil percentage to albumin ratio (NPAR) were independently associated with ARF onset. The concordance index of the nomogram incorporating these predictors was 0.933 in the training cohort and 0.955 in the test cohort, although both calibrations were good. The AUC of the nomogram was equal to concordance index, which presented better predictive accuracy compared with previous measurements using neuroimaging and AIS grade (AUC 0.933 versus 0.821, Delong’s test *p* < 0.001). Similar significant results were also found in the test cohort (AUC 0.955 versus 0.765, Delong’s test *p* = 0.034). In addition, this nomogram was translated to a Web-based calculator that could generate individual probability for ARF in a visualized form.

**Conclusions:**

The nomogram incorporating the injury level, AIS grade, admission Hb, platelet to lymphocyte ratio, and NPAR is a promising model to predict ARF in patients with cervical TSCI who are absent from previous respiratory dysfunction. This nomogram can be offered to clinicians to stratify patients, strengthen evidence-based decision-making, and apply appropriate individualized treatment in the field of acute clinical care.

**Supplementary Information:**

The online version contains supplementary material available at 10.1007/s12028-021-01302-4.

## Introduction

Patients who sustain cervical TSCI commonly experience respiratory complications [1, 2]. ARF favored by progressive myopathies of bulbar and expiratory muscle, serves as one of leading causes that culminates in poor prognosis or even death [3], which compromise the life expectancy for a patient with cervical TSCI.

Injury level and severity to the cervical cord are considered main factors related to the development of respiratory complications, including ARF [4, 5]. These parameters are regularly assessed by neuroimaging tools and the American Spinal Injury AIS in clinical practice [6, 7]. There is also evidence showing that the presence of chronic respiratory diseases in patients with TSCI is clinically associated with an increased risk of respiratory failure [8]. It has been a consensus for the clinicians that the ARF is quite frequent among patients with premorbid history of respiratory diseases, and the early introduction of proactive ventilatory managements, such as endotracheal intubation, should be fully considered based on evaluations [9]. However, clinicians often hesitate or even fail to perform appropriate assistance to prevent ARF in the patients who are lacking the evidence of enduring chronic respiratory diseases because of the difficulty in predicting the development of ARF by using current clinical approaches. Meanwhile, the sudden and precipitous functional defects in the respiratory muscle caused by the cervical TSCI further add complexity to the accurate prediction [6]. In this context, the accurate prediction of ARF is pivotal to clinical practice and, ultimately, to improving clinical outcome following cervical TSCI.

Over the past decades, numerous efforts have been devoted to identifying risk factors to predict complications and outcomes following cervical TSCI. It is believed that inflammatory response is the major pathogenic mechanism that is swiftly activated at the time of primary injury and subsequently mediates secondary injury within acute phase [10, 11]. As the major immune components, neutrophils, lymphocytes, and monocytes were reported to gradually infiltrate into the lesion site post primary injury, participating in multiple pathophysiological processes [12–14]. Inflammatory indices, such as neutrophil to lymphocyte ratio (NLR), platelets to lymphocyte ratio (PLR), or systemic inflammation response index (SIRI), have emerged to play significant roles in predicting clinical outcome of neurotraumatic diseases, including TSCI [15]. Meanwhile, the majority of patients with cervical TSCI are healthy individuals with a normal nutritional status prior to injury [16] and may thus be adversely affected during acute stage [17] with metabolic changes, including reduced basal energy expenditure, increased nitrogen excretion, and negative changes of several nutritional indicators [18]. Plasma levels of nutritional components, such as serum albumin or creatinine, were decreased in patients of TSCI during acute phase [17, 19, 20]. Besides, these biochemical markers were able to interact with inflammation reaction [21]. Because respiratory muscle are susceptible to the imbalance of inflammatory condition and altered nutritional status [22], we assumed the markers of inflammation and altered nutrition might contribute to the early prediction of ARF following cervical TSCI. However, very few validated models based on the inflammation and altered nutrition parameters have been established for predicting the onset of ARF secondary to cervical TSCI.

Based on these considerations, this study retrospectively reviewed clinical data of patients with cervical TSCI who developed ARF but without chronic respiratory diseases. We aimed to investigate the novel risk factors of ARF after primary injury of cervical TSCI. In addition to the conventional neuroimaging and AIS evaluation methods, our model was based on the inflammatory and altered nutritional parameters on admission to predict the risk of ARF occurrence following cervical TSCI with external validation.

## Methods

### Study Population

This study was conducted on two independent cohorts of patients. We retrospectively reviewed the electronic medical data from patients with TSCI enrolled in our institution between January 1, 2014, and September 30, 2020, as the training cohort. The institutional review board was approved by the Ethnic Committee of Qingdao Municipal Hospital. The inclusion criteria were as follows: (1) cervical TSCI; (2) 18 years old or above; (3) admitted to our institution within 48 h post primary injury; (4) undergoing rehabilitation at our institution; and (5) complete data with the baseline blood, neuroimaging and neurological (AIS) assessments. Patients were considered ineligible if they suffered (1) severe concomitant traumatic cerebral injuries; (2) TSCIs related to other non‐traumatic pathologies such as infection or tumor; (3) a history of previous stroke or dementia that leads to noncooperation with neurological evaluation; (4) a history of chronic respiratory diseases; (5) prior systemic diseases such as renal failure, malignancy, or liver cirrhosis; (6) incomplete or missing information. For external validation of the model, patients admitted to Binzhou Medical University Hospital between January 1, 2014, and January 31, 2021, with cervical TSCI were retrospectively collected as the test cohort in accordance with the inclusion and exclusion criteria. The review board was also discussed and approved by the by the Ethnic Committee of Binzhou Medical University Hospital.

### Clinical Variables Collection

The clinical variables were collected as follows:

1. Demographic data: age, sex, and smoking status.
2. Premorbid history: hypertension, chronic heart disease, and diabetes status.
3. Baseline characteristics of cervical TSCI: systolic blood pressure (SBP), diastolic blood pressure (DBP), heart rate (HR), causes of cervical TSCI, the level of injury, and AIS classification on admission.
4. Surgical options for patients with cervical TSCI.

Presence of ARF was defined as an arterial oxygen saturation of < 90% or a partial pressure of arterial oxygen of < 60 mm Hg while breathing room air, and/or respiratory acidosis (pH < 7.35 with a partial pressure of arterial carbon dioxide > 45 mm Hg).

### Laboratory Parameters

Inflammatory markers, including white blood cell (WBC), neutrophil count (NC), lymphocyte count (LC), monocyte count (MC), and platelets volume, were extracted from emergent test of complete blood count. The NLR, PLR, and SIRI (defined as NC × MC/LC) were calculated.

For nutritional variables collection, according to prior study [23], a series of biomedical markers, including Body Mass Index (BMI), Hb, serum creatinine (SCr), red cell distribution width (RDW), albumin, and prealbumin, were determined to be reflective of nutritional status. Further, we took NPAR into analysis, as it is a newly indicator for prognosis combing both inflammatory and nutritional parameters in several critical illness and may serve predictive value in cervical TSCI.

### Clinical Features Following ARF Occurrence and In-Hospital Mortality

Patients who were diagnosed ARF were regularly transferred to intensive care unit (ICU) for special medical care. The time from primary injury to ARF occurrence, use of medical interventions (such as tracheostomy and length of mechanical ventilation), length of ICU stays, severe complications, and surviving patients with ARF were also assessed.

### Statistical Analysis and Predictive Model Creation

Categorical variables were represented as percentages. Continuous variables were represented as mean ± standard deviation or median with the interquartile range. The comparisons of continuous variables were performed using the *t*-test for normally distributed or the Mann–Whitney *U*-test or nonnormally distributed continuous variables checked by Kolmogorov–Smirnov statistics, while *χ*^2^ test or Fisher’s exact test was used for categorical variables. Univariate analysis was conducted to test the impact of admission risk factors for ARF occurrence. A forward stepwise multivariate logistic regression analysis was used to investigate independent predictors of ARF occurrence, which included variables with *p* < 0.2 from the univariate analysis. Odds ratio (OR) with 95% confidence interval (CI) were represented as results. The variance inflation factor (VIF) and tolerance were used to check multicollinearity between variables.

We constructed receiver operative curve (ROC) and the area under the curve (AUC) to measure the specificity and sensitivity of models and their discriminative power respectively, with values between 0.90 and 1.00 indicative of excellent predictive discrimination [24]. To promote model visualization, a nomogram based on independent predictors from multivariate logistic regression model was established using R 4.0.1 for Windows*. DynNom* and *Shiny* packages were exploited to generate an online calculator (https://www.shinyapps.io/). The discriminative ability of nomogram was tested by the concordance index (C-index), and the 1000-repetition bootstrap resampling strategy was used for optimistic correction. The calibration was confirmed by the Hosmer–Lemeshow test. All statistical tests were two-tailed and *p* values < 0.05 were considered statistically significant.

## Results

### Demographic Data and Clinical Features

**Fig. 1:**
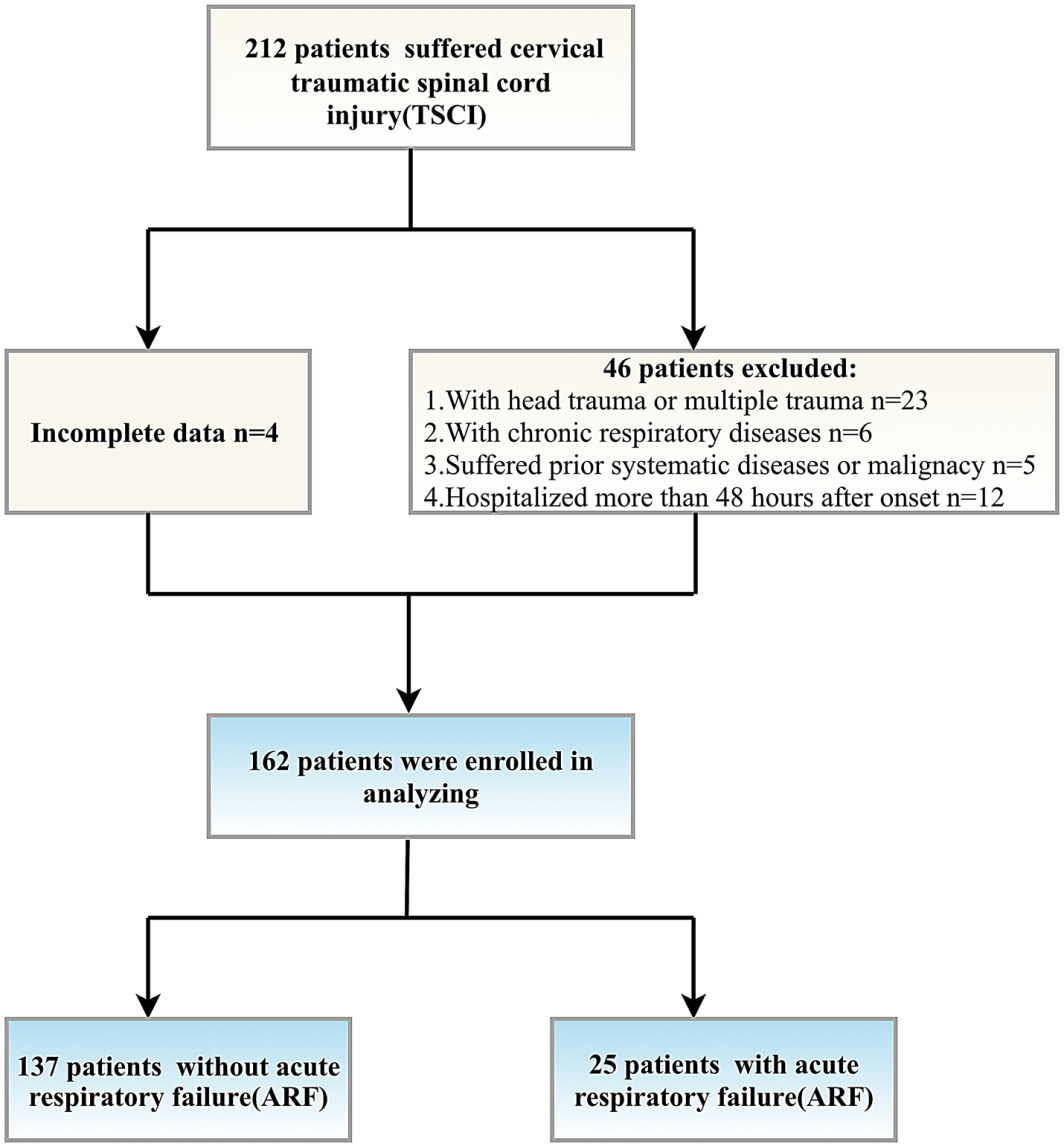
Diagram of study design. ARF, acute respiratory failure

In the training cohort, 212 patients admitted to Qingdao Municipal Hospital between 2014 and 2020 with the primary diagnosis as cervical TSCI, of whom a total of 162 patients were considered eligible for further analysis (Fig. 1). Baseline characteristics are displayed in Table 1. Mean age at the time of injury was 58.9 ± 12.5 years, with the majority of patients being men (75.3%). The most common etiology of cervical injury was falling (56.8%), followed by traffic accidents (37.7%), sports-related accidents (3.7%), and other causes (1.9%), such as suicide or occupational damage. Most patients were clinically diagnosed as incomplete injury of spinal cord injury (SCI) with an AIS grade of C or D (82.7%). After admission, 117 (72.2%) patients were surgically treated within acute or subacute phase, whereas 45 (27.8%) received consecutive conservative treatments.

**Table 1:** Comparison of demographic, clinical and laboratory characteristics of patients with cervical TSCI according to the onset of ARF in training cohort

Variable	Total	Without ARF	With ARF	*p* value
Age (yr)^a^	58.90 ± 12.52	59.01 ± 12.63	58.28 ± 12.15	0.790
Sex^*b*^				0.554
Male	122 (75.3)	102 (74.5)	20 (80.0)	
Female	40 (24.7)	35 (25.5)	5 (20.0)	
Cause of TSCI^*b*^				0.078
Fall	92 (56.8)	79 (57.7)	13 (52.0)	
Traffic accidents	61 (37.7)	53 (38.7)	8 (32.0)	
Sports-related accident	6 (3.7)	3 (2.2)	3 (12.0)	
Other causes	3 (1.9)	2 (1.5)	1 (4.0)	
Premorbid history^*b*^				
Hypertension	46 (28.4)	36 (26.3)	10 (40.0)	0.162
CHD	20 (12.3)	18 (13.1)	2 (8.0)	0.742
Diabetes	26 (16.0)	20 (14.6)	6 (24.0)	0.243
Smoker	39 (24.1)	33 (24.1)	6 (24.0)	0.992
Admission characteristics				
SBP (mm Hg)^a^	133.31 ± 19.66	133.88 ± 19.00	130.16 ± 23.14	0.386
DBP (mm Hg)^c^	79.00 (70.00, 85.25)	79.00 (70.00, 85.00)	75.00 (61.00, 87.50)	0.237
HR (beats/min)^c^	72.00 (68.00, 80.00)	72.00 (68.00, 80.00)	72.00 (66.50, 80.00)	0.711
Level of SCI^*b*^				0.003
C4–C1	66 (40.7)	49 (35.8)	17 (68.0)	
C4–C7	96 (59.3)	88 (64.2)	8 (32.0)	
Combined with thoracic trauma^*b*^				0.716
Yes	16 (9.9)	13 (8.0)	3 (1.9)	
No	146 (90.1)	124 (76.5)	22 (13.6)	
Type of SCI^*b*^				< 0.001
Complete	24 (14.8)	10 (7.3)	14 (56.0)	
Incomplete	138 (85.2)	127 (92.7)	11 (44.0)	
AIS grade^*b*^				< 0.001
A or B	28 (17.3)	12 (8.8)	16 (64.0)	
C or D	134 (82.7)	125 (91.2)	9 (36.0)	
Treatment^*b*^				0.142
Anterior surgery	64 (39.5)	58 (42.3)	6 (24.0)	
Posterior surgery	53 (32.7)	41 (29.9)	12 (48.0)	
Conservative	45 (27.8)	38 (27.7)	7 (28.0)	
Laboratory indices				
WBC (10^9^/L)^c^	8.75 (6.45, 11.23)	8.51 (6.37, 11.06)	10.24 (7.84, 12.15)	0.078
NC (10^9^/L)^c^	6.71 (4.98, 9.47)	6.22 (4.66, 9.22)	8.79 (6.60, 10.64)	0.009
LC (10^9^/L)^c^	1.15 (0.82, 1.74)	1.24 (0.89, 1.84)	0.82 (0.59, 1.16)	< 0.001
MC (10^9^/L)^c^	0.35 (0.13, 0.58)	0.35 (0.15, 0.58)	0.30 (0.06, 0.58)	0.503
Platelets (10^9^/L)^c^	200.50 (166.00, 250.25)	201.00 (168.00, 251.05)	190.0 (160.00, 235.50)	0.307
Scr (μmol/L)^c^	65.60 (54.13, 72.74)	66.08 (54.69, 72.83)	66.80 (50.27, 68.82)	0.234
BMI (kg/m^2^)^c^	24.47 (22.65, 26.24)	24.22 (22.59, 26.26)	25.18 (23.89, 26.12)	0.206
Hb (g/L)^c^	138.00 (126.00, 149.25)	139.00 (130.00, 150.50)	124.00 (109.00, 138.50)	< 0.001
RDW (fl)^c^	43.25 (41.30, 45.40)	43.30 (41.35, 45.15)	42.80 (40.90, 46.45)	0.926
Albumin (g/dL)^a^	3.78 ± 0.50	3.85 ± 0.46	3.38 ± 0.52	< 0.001
Prealbumin (mg/L)^c^	257.00 (211.00, 303.00)	259.00 (211.00, 306.50)	241.00 (214.00, 268.00)	0.201
NPAR^c^	20.78 (17.94, 23.69)	20.10 (17.19, 22.84)	25.56 (22.64, 28.26)	< 0.001
NLR^c^	5.93 (3.17, 10.35)	5.20 (2.63, 9.44)	10.67 (6.05, 16.60)	< 0.001
SIRI^c^	1.37 (0.64, 3.18)	1.32 (0.62, 3.15)	6.10 (2.38, 12.09)	0.079
PLR^c^	165.30 (117.78, 234.21)	152.94 (111.93, 216.97)	235.42 (168.59, 393.96)	0.001

AIS, American Spinal Injury Association Impairment Scale, ARF, acute respiratory failure, BMI, Body Mass Index, CHD, chronic heart disease, DBP, diastolic blood pressure, Hb, hemoglobin, HR, heart rate, LC, lymphocyte count, MC, monocyte count, NC, neutrophil count, NLR, neutrophil to lymphocyte ratio, NPAR, neutrophil percentage to albumin ratio, PLR, platelet to lymphocyte ratio, RDW, red cell distribution width, SBP, systolic blood pressure, SCI, spinal cord injury, Scr, serum creatinine, SD, standard deviation, SIRI, systemic inflammation response index, TSCI, traumatic spinal cord injury, WBC, white blood cell

^a^Mean ± SD

^b^Percentage (%)

^c^Median (25th, 75th)

Of these patients, 26 (15.4%) developed ARF. As shown in Table 2, the median time of ARF occurrence was 3 days post primary injury. Mechanical ventilation was supplied to reverse hypoxemia and prevent respiratory complications immediately after the ARF onset. Fifteen patients (60%) received tracheostomy after the consensus of at least two senior clinicians for enhanced mechanical ventilation. Despite the intensive medical support, the majority of these patients eventually developed pneumonia (72%) or severe organ complications, including acute respiratory distress syndrome (56%), coagulopathy (28%), and renal dysfunction (20%). The median length of ICU cares was 12 days, and 14 individuals (56%) died during this period. In contrast, only two patients died in hospitalization of non-ARF-related complications.

**Table 2:** Clinical features of patients after ARF onset

Variables	Values
Time of ARF, median (IQR) (d)	3.0 (2.0, 8.0)
Complications during ICU stay, n (%)	
Pneumonia	18 (72.0)
Acute respiratory distress syndrome	14 (56.0)
Renal dysfunction	5 (20.0)
Coagulopathy	7 (28.0)
ICU length of stay, median (IQR) (d)	13.0 (3.5, 30.0)
Tracheostomy, *n* (%)	15 (60.0)
Length of mechanical ventilation, median (IQR) (d)	12.0 (5.0, 29.5)
Died in the hospital, *n* (%)	14 (56.0)

ARF, acute respiratory failure, ICU, intensive care unit, IQR, interquartile range

The univariate analysis revealed risk factors of ARF using characteristics at the time of admission. Patients with high level of cervical injury (C1–C4) and AIS grading of A or B had high risk of ARF occurrence (*p* = 0.003, *p* < 0.01 respectively). Other demographic variables, including sex, premorbid history of hypertension or chronic heart diseases, smoking status, diabetes, causes of primary injury, measurements of blood pressure and HR, and the types of treatments, showed no statistical relationship with ARF. For laboratory tests, such patients with ARF were recorded to have higher admission WBC, NC, NLR, PLR, and SIRI, but lower levels of LC, MC, and platelets volume. Meanwhile, undernutrition status was generally observed in patients with lower admission levels of Hb, RDW, albumin, and prealbumin. NPAR as a marker combining parameters from complete blood count and biochemistry analysis was also found to be higher in patients with ARF. These blood variables were introduced in the multivariate analysis, along with injury level and AIS grades.

### Predictors for ARF and Model Development

**Fig. 2:**
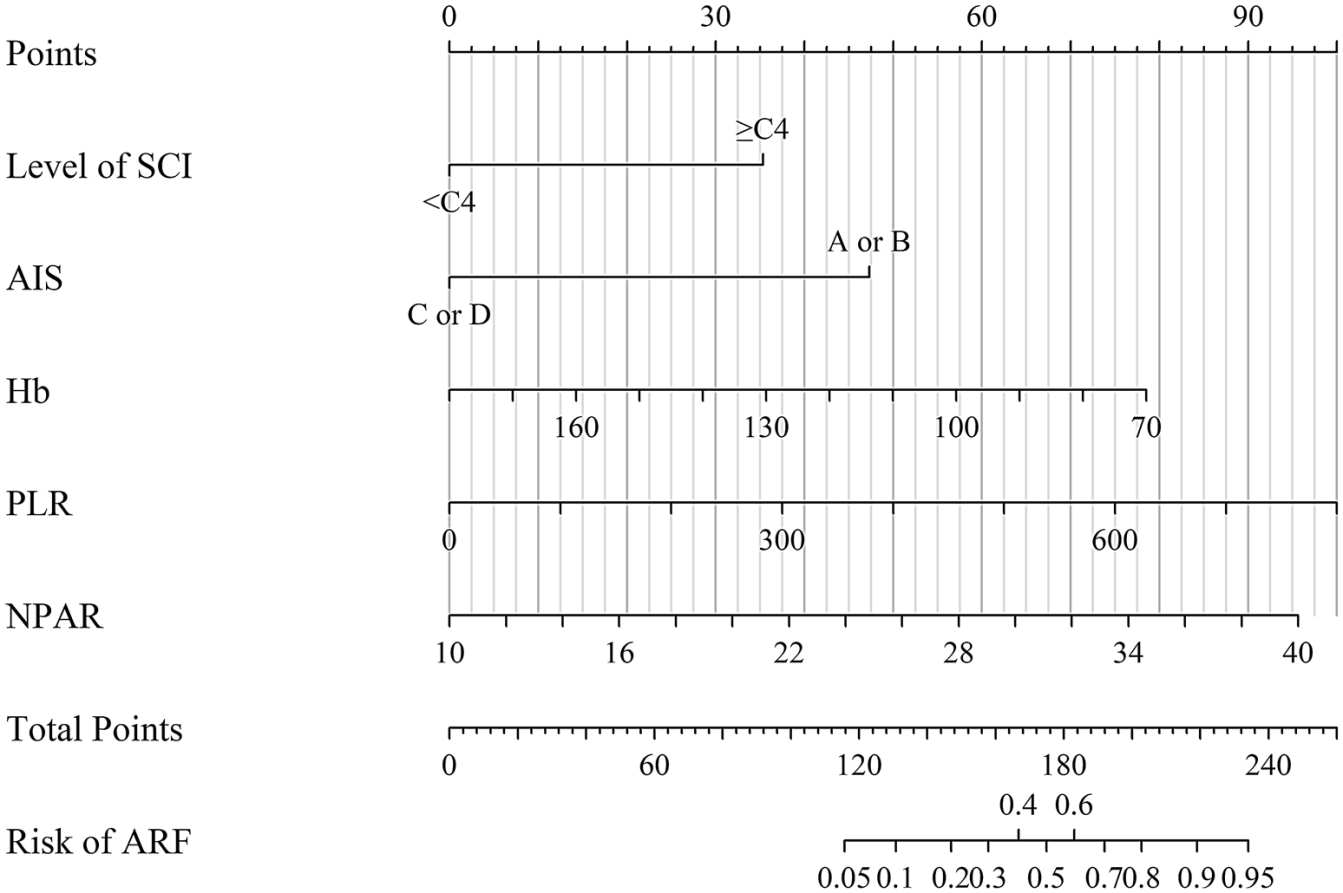
Nomogram for predicting ARF after cervical TSCI in training cohort. Five independent predictors were involved in this mode and each of them was assigned with a graphic score. The sum of these five scores generated a plot on “total points” axis. The individual probability of ARF occurrence was summarized by drawing a vertical line from the “Total points” axis to “Risk” axis. ARF, acute respiratory failure, TSCI, traumatic spinal cord injury

Table 3 shows the independent predictors of ARF. Finally, after multivariable adjustment, five predictors remained significant in multivariate logistic regression analysis. Injury level above C4 (OR = 5.796, 95% CI 1.646–20.404), grade A or B of AIS (OR = 10.540, 95% CI 2.995–37.090), lower Hb (OR = 0.965, 95% CI 0.936–0.995), and higher PLR (OR = 1.006, 95% CI 1.002–1.011), as well as NPAR (OR = 1.172, 95% CI 1.022–1.344) predicted an increased risk of ARF. Multicollinearity diagnostic tests showed that the tolerance was > 0.5 and the VIF was < 10 for all the predictors (Supplementary Table 1). Thus, a model including these five independent predictors was developed to predict the onset of ARF in patients with cervical TSCI. We also constructed a nomogram, in which a graphic score was statistically assigned to each of the significant predictors for quick reference (Fig. 2). The total points of summed scores referred to an individual probability of ARF after cervical TSCI. We evaluated the discrimination of the nomogram by calculating C-index, which was 0.933, with optimistic correction by 1,000 repetitions bootstrap strategy.

**Table 3:** Multivariate logistic regression analysis for predictors of ARF development using variables known at the time of admission

Risk factors	B	Standard error	Wald	*p* value	OR	95% CI of OR
Level of TSCI (C4–C1)	1.757	0.642	7.488	0.006	5.796	1.646–20.404
AIS (A or B)	2.355	0.642	13.461	< 0.001	10.540	2.995–37.090
Hb	− 0.036	0.016	5.052	0.025	0.965	0.936–0.995
PLR	0.006	0.002	8.080	0.004	1.006	1.002–1.011
NPAR	0.159	0.070	5.178	0.023	1.172	1.022–1.344
Constant	− 3.895	2.671	2.127	0.145	0.02	

AIS, American Spinal Injury Association Impairment Scale, ARF, acute respiratory failure, B, regression coefficient, CI, confidence interval, Hb, Hemoglobin, NPAR, neutrophil percentage to albumin ratio, OR, odds ratio, PLR, platelet to lymphocyte ratio, TSCI, traumatic spinal cord injury

**Fig. 3:**
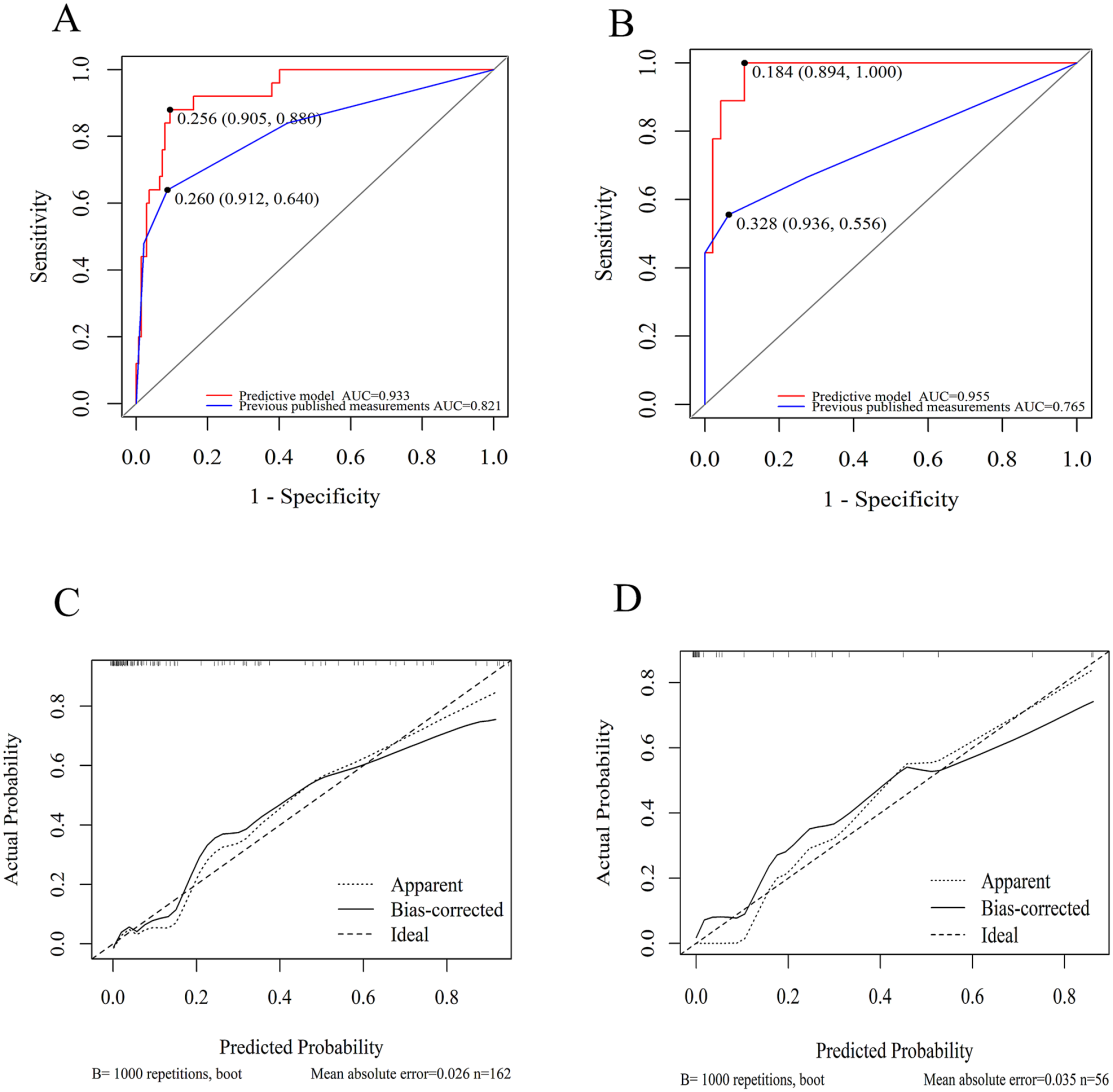
Discriminability and calibration curves of nomogram. Discriminability between newly developed nomogram and previous published measurements was compared with ROC analysis in training cohort (**a**) and test cohort (**b**). The cut-off value of nomogram and traditional measurements for predicting in training cohort was 25.6% (sensitivity 90.5%, specificity 88.0%) and 26% (sensitivity 91.2%, specificity 64.0%) while values in test cohort was 18.4%(sensitivity 89.4%, specificity 100.0%) and 32.8% (sensitivity 93.6%, specificity 55.6%) respectively. AUC of the nomogram showed significant enhancement in contrast to traditional measurements only composed injured level and AIS grade (0.933 versus 0.821, *p* < 0.001) in training cohort and (0.955 versus 0.765, *p* = 0.034) in test cohort. Calibration curve presented prediction of ARF onset between the nomogram prediction and actual observation. Hosmer–Lemeshow test indicated good prediction of our nomogram (*p* = 0.686) (**c**), similarly in the test cohort (*p* = 0.291) (**d**). AIS, American Spinal Injury Association Impairment Scale, AUC, area under the receiver operating characteristic curve, ROC, receiver operating characteristic curve

ROC curves regarding the discriminatory power of the nomogram and the previous published measurements are shown in Fig. 3a. Compared with the predictive tools using radiological and AIS data (AUC 0.821, 95% CI 0.715–0.927), the results of DeLong’s test (Supplementary Table 2) indicated significant improvement for the new nomogram (AUC 0.933, 95% CI 0.887–0.980, *p* < 0.001). Figure 3c reveals the calibration plot comparing the prediction of ARF between the nomogram prediction and actual observation. Following Hosmer–Lemeshow test (*p* = 0.686), the calibration plot proved good predictive accuracy of this nomogram.

### External Validation of the Nomogram

We performed a geographical validation by employing an independent cohort (Supplementary Table 3), in which 56 patients were retrospectively enrolled. According to this data set, a total of nine patients who experienced ARF were eligible for model validation (Table 4). In Fig. 3b, based on DeLong’s test (*p* = 0.034), the AUC of the newly nomogram in the test cohort (AUC 0.955, 95% CI 0.905–0.991), which equated to C-index, was also superior to previous published measurements (AUC 0.765, 95% CI, 0.554–0.975). The calibration plots exhibited an acceptable agreement in the test cohort (Hosmer–Lemeshow test, *p* = 0.291; Fig. 3d).

**Table 4:** Clinical features of the external validation group

Risk factors	Total (*n* = 56)	Without ARF (*n* = 47)	With ARF (*n* = 9)
Level of SCI			
C4–C1	15 (26.8)	10 (21.3)	5 (55.6)
C4–C7	41 (73.2)	37 (78.7)	4 (44.4)
AIS			
A or B	8 (14.3)	3 (6.4)	5 (55.6)
C or D	48 (85.7)	44 (93.6)	4 (44.4)
Hb	136.00 (123.50, 146.00)	138.00 (130.00, 147.00)	123.00 (106.50, 129.50)
PLR	176.58 (119.61, 271.95)	174.29 (117.06, 253.23)	347.50 (118.93, 546.67)
NPAR	20.65 (18.35, 23.39)	20.17 (16.90, 21.90)	25.60 (23.84, 26.83)

ARF, acute respiratory failure, AIS, American Spinal Injury Association Impairment Scale, Hb, Hemoglobin, NPAR, neutrophil percentage to albumin ratio, PLR, platelet to lymphocyte ratio, SCI, spinal cord injury

### Online Calculator for Generating Risk of ARF Occurrence

**Fig. 4:**
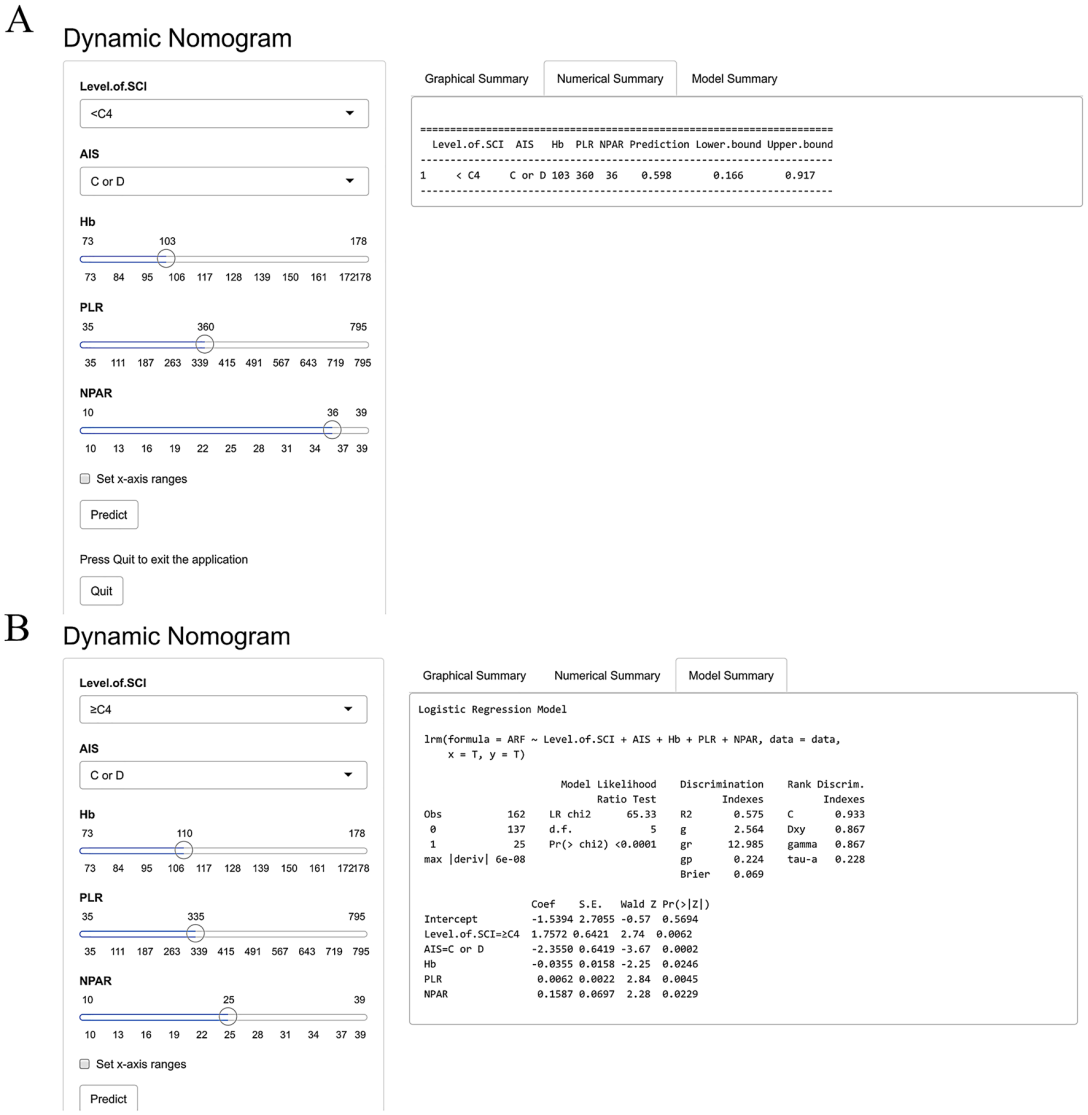
The online calculator translated from nomogram for generating risk of ARF onset. **a** Numerical summary of prediction. **b** Model details of prediction. ARF, acute respiratory failure

A dynamic Web-based calculator was created according to the results above (the access link is https://dynomogramsciqdmh.shinyapps.io/DynNomapp/). For example, assume there was a patient with a lower level of injury (below C4) identified by neuroimaging and their AIS grade was C, PLR value was 360, NPAR value was 36, and admission Hb concentration was 103 g/L; the probability of ARF occurrence in this patient would be 59.8% (95% CI 16.6–91.7) based on our model (Fig. 4a, b).

## Discussion

ARF is a devastating event after traumatic injuries to the cervical spine, and the triggers for ARF are multifactorial. This study found that the five variables collected on admission (injured level of cervical spine, AIS grades, Hb concentration, PLR, and NPAR) were significantly related to ARF, based on the logistic regression analysis. To our best knowledge, this is the first study establishing a prediction model for evaluating the risk of ARF in patients with cervical TSCI, especially for those without history of respiratory diseases.

Once the ARF emerged, patients were going to develop severe organ complications, receive more intensive treatments (mechanical ventilation or tracheostomy) and prolonged ICU stay. In the current study, 26 individuals developed ARF and 14 of them died despite ICU stay, which further demonstrated the devastating outcome related to the ARF. Clinically, the requirement for urgent assistance to prevent respiratory failure in patients with SCI is determined by the spine level and the severity of primary injury [25]. The current practice for the ARF prediction in the first 72 h post injury is to combine the neuroimaging and AIS examination results [26]. However, the reliability of this method was poor because of the lack of SCI expertise or the challenge to accurately evaluate the patients with SCI from various primary injuries [26, 27]. This being the case, the quicker and more accurate the prediction of ARF is, the more likely the patients will benefit from appropriate clinical supports.

Recent publications highlighted that the biomarkers are clinically useful to predict complications or prognosis in severe illness but, in fact, there is variability in the length of time it takes to attend the emergency department and further variability in the time it takes to perform clinical examinations. As a result, various time points were described to withdraw biomarkers for analysis. Wilson and his colleagues [24] have addressed the importance of the data obtained during the initial injury period (within 3 days post primary SCI), during which there is a significant demand for acute therapeutic decision. Because the median time of ARF occurrence observed was 3 days after cervical SCI, we assumed that the ARF onset might be significantly affected by the pathophysiological changes during acute phase, and it was realistic to include the admission biomarkers from patients who emergently attended to our center within 48 h post primary injury for analysis. In this period, acute inflammatory response was activated around the lesion site and, subsequently, the inflammatory cytokine levels increased [28]. Moreover, it should be noted that the additional metabolic and nutritional deficiencies exist among nearly two thirds of individuals with SCI, which could cause respiratory muscle atrophy [29, 30] and generate high risk of respiratory exhaustion. Further, accumulated evidence has shown that the nutritional status is significantly perturbated by the presence of inflammatory reaction in acute illness [31, 32]. Hence, the circulating biomarkers reflecting the inflammation and malnutrition levels on admission are promising to facilitate early prediction of ARF in patients with cervical TSCI. Kwon et al. [33] and Yang et al. [34] have discovered that several inflammatory markers were elevated in cerebrospinal fluid or serum of patients with acute SCI, and serum albumin is a classic biomedical indicator for evaluating nutritional status [29]. However, the translational use of these markers is weak in clinical practice. Ideal laboratory biomarkers are described as less invasive, stably reproducible, and easy to detect. Detecting specific markers are technically demanding, time consuming, and, to some extent, not as cost-effective as some laboratory reagents or kits are required. In addition, the role of serum albumin as an indicator of nutritional status has also been largely questioned and diminished [31]. So, it is doubtful for a clinician to use such results to make prediction and take correct medical decisions in acute phase.

Many previous studies suggested a combination of biomarkers to predict complications or outcomes of TSCI. However, given the clinical heterogeneity of patients with TSCI and the lack of variables stratification, it is natural to question whether such combination would better predict the ARF risk than the traditional gold standard (neuroimaging and neurological grading). In response to this, we had interests in investigating biomarkers from complete blood count and biochemistry analysis to build a predictive model for ARF because they are routinely performed at the time of admission for each patient. These tests are low-cost, and abnormalities were simply obtained within few hours. We developed a predictive nomogram incorporating clinical gold standard (neuroimaging + AIS) and independent markers from routine blood tests. Compared with the predictive values of gold standard, our nomogram showed better discrimination and calibration verified by DeLong’s and Hosmer–Lemeshow tests, respectively. Thus, we believed this nomogram could serve as an effective tool to help clinicians to identify patients with cervical TSCI at increased risk of ARF on admission and choose the appropriate therapeutic plan.

Although the inflammatory indices like neutrophil, lymphocyte, NLR, and SIRI showed difference between patients with and without ARF, only the high PLR was identified independently associated with increased risk of ARF by the collinearity assessment and multivariate logistic regression analysis. Platelets mediate strong inflammation by releasing multiple cytokines [35]. Increased PLR, indicating high volume of platelets and low LCs, could reflect the burden of plaques in atherosclerosis [36] and the infarct size of acute ischemic stroke [37]. Meanwhile, we found that the patients with cervical TSCI with elevated NPAR had a significantly higher risk of ARF. As a newly discovered biomarker composed of neutrophil percentage and albumin level, NPAR has been shown promising in predicting the prognosis of coronary artery disease [38, 39]. For nutritional assessment, results that serum albumin or prealbumin showed no significant correlation was not surprising. We found low Hb concentration was remarkably associated with ARF risk. Physiologically, Hb delivers oxygen to tissue. Abundant Hb concentration was efficient to prevent hypoxia and cell energy disruption [40]. Interestingly, these three predictors identified above were all correlated to vascular diseases and importantly, ischemic events. Taken together, we hypothesized that during acute period of cervical TSCI, microischemic events were potentially triggered in response to inflammatory changes as well as malnutrition status, then hypoxic damage was activated around the lesion site, affecting the spinal nerves that innervate respiratory muscles and ultimately resulting in ARF. Although thoracic trauma was addressed as an independent risk factor for respiratory complications in SCI [8], our data revealed that the coexistence of thoracic trauma during acute admission period was not significantly correlated to the ARF occurrence in patients with cervical TSCI, which was similar to the previous study [6]. We supposed that the impacts of high cervical injuries overweighted thoracic trauma in inducing ARF within acute period because muscles of inhalation and exhalation were mostly innervated by the phrenic motoneurons located between midcervical cord [41], and injuries to these segments were the most prevalent form of trauma that led to respiratory dysfunction [42], compared with other thoracic regions where there are no key muscles [43]. Nevertheless, studies with further stratifications are needed to investigate the roles of thoracic trauma in inducing ARF occurrence in both acute and rehabilitation stages. To make this predictive model more user-friendly, an online calculator has been developed, which instantly calculates the ARF risk after clinicians type in the specific parameters, making it easily applicable in clinical practice.

There are several advantages in our study. (1) It is the first nomogram designed to predict ARF occurrence in the patients with cervical TSCI, especially for those without previous respiratory diseases. (2) The predictors of our nomogram were routinely measured and available for each patient on admission. (3) Given our data obtained within the acute period for all individuals with cervical TSCI, this nomogram is going to provide individualized and precision therapeutic strategies as it can identify patients at high risk of ARF as soon as clinicians expected. Meanwhile, we also recognize several limitations. (1) The statistical analysis in the current study might not strictly meet the principle of events per variable due to the limit of ARF occurrence in small sample size, even though we used retrospective data from another medical center to perform an external validation. (2) Several parameters including the erythrocyte sedimentation rate, C-reactive protein, iron, transferrin, or vitamins may not be regularly measured in the admission blood tests, which might compromise the statistical validity of our model. A larger data set from multiple centers is strongly recommended to provide robust evidence to predict the risk of ARF. (3) Only the patients encountered the traumatic etiology of SCI were taken into analysis which may be a limitation in extending our model to other affected individuals. (4) Because we aimed at providing an instant prediction within the acute admission period, risk factors that significant contribute to ARF in rehabilitation stage including pneumonia or thoracic trauma were not fully taken into analysis.

## Conclusions

ARF as a severe respiratory complication could suddenly occur in patients who suffered cervical TSCI, even though they have no previous respiratory diseases. Knowledge of characteristics in the realm of acute clinical care, including injury cervical level, AIS grade, blood Hb concentration, PLR, and NPAR, can effectively help to identify patients at high risk of developing ARF following cervical TSCI. Early prediction of ARF occurrence using nomogram composed of these factors is expected to guide clinicians to provide personalized supports and nursing strategies.

## Supplementary Information

Below is the link to the electronic supplementary material.

Supplementary file 1 (DOCX 16 kb)

Supplementary file 2 (DOCX 20 kb)

Supplementary file 3 (XLSX 59 kb)

Supplementary file 4 (XLSX 11 kb)

Supplementary file 5 (XLSX 21 kb)

## References

[1] van den Berg MEL, Castellote JM, de Pedro-Cuesta J, Mahillo-Fernandez I (2010). Survival after spinal cord injury: a systematic review. J Neurotrauma.

[2] Berlowitz DJ, Tamplin J. Respiratory muscle training for cervical spinal cord injury. Cochrane Database Syst Rev. 2013;CD008507. 10.1002/14651858.CD008507.pub2 PMC11089516 23881660

[3] Ganuza JR, Forcada AG, Gambarrutta C (2011). Effect of technique and timing of tracheostomy in patients with acute traumatic spinal cord injury undergoing mechanical ventilation. J Spinal Cord Med.

[4] Linn WS, Adkins RH, Gong H, Waters RL (2000). Pulmonary function in chronic spinal cord injury: a cross-sectional survey of 222 Southern California adult outpatients. Arch Phys Med Rehabil.

[5] Zakrasek EC, Nielson JL, Kosarchuk JJ, Crew JD, Ferguson AR, McKenna SL (2017). Pulmonary outcomes following specialized respiratory management for acute cervical spinal cord injury: a retrospective analysis. Spinal Cord.

[6] Aarabi B, Harrop JS, Tator CH (2012). Predictors of pulmonary complications in blunt traumatic spinal cord injury. J Neurosurg Spine.

[7] Stein DM, Menaker J, McQuillan K, Handley C, Aarabi B, Scalea TM (2010). Risk factors for organ dysfunction and failure in patients with acute traumatic cervical spinal cord injury. Neurocrit Care.

[8] Sampol J, Gonzalez-Viejo MA, Gomez A (2020). Predictors of respiratory complications in patients with C5–T5 spinal cord injuries. Spinal Cord.

[9] Racca F, Vianello A, Mongini T (2020). Practical approach to respiratory emergencies in neurological diseases. Neurol Sci.

[10] Capirossi R, Piunti B, Fernandez M, et al. Early CSF Biomarkers and late functional outcomes in spinal cord injury. A pilot study. Int J Mol Sci. 2020;21. 10.3390/ijms21239037 PMC7729583 33261156

[11] Albayar AA, Roche A, Swiatkowski P, et al. Biomarkers in spinal cord injury: prognostic insights and future potentials. Front Neurol. 2019;10. 10.3389/fneur.2019.00027 PMC6361789 30761068

[12] Neirinckx V, Coste C, Franzen R, Gothot A, Rogister B, Wislet S. Neutrophil contribution to spinal cord injury and repair. J Neuroinflammation. 2014;11. 10.1186/s12974-014-0150-2 PMC4174328 25163400

[13] Jenne CN, Kubes P (2015). Platelets in inflammation and infection. Platelets.

[14] Beck KD, Nguyen HX, Galvan MD, Salazar DL, Woodruff TM, Anderson AJ (2010). Quantitative analysis of cellular inflammation after traumatic spinal cord injury: evidence for a multiphasic inflammatory response in the acute to chronic environment. Brain.

[15] Zhao JL, Lai ST, Du ZY, et al. Circulating neutrophil-to-lymphocyte ratio at admission predicts the long-term outcome in acute traumatic cervical spinal cord injury patients. BMC Musculoskelet Disord. 2020;21. 10.1186/s12891-020-03556-z PMC7429795 32799840

[16] Chen X, Liu Z, Sun T, Ren J, Wang X (2014). Relationship between nutritional status and mortality during the first 2 weeks following treatment for cervical spinal cord injury. J Spinal Cord Med.

[17] Laven GT, Huang CT, DeVivo MJ, Stover SL, Kuhlemeier KV, Fine PR (1989). Nutritional status during the acute stage of spinal cord injury. Arch Phys Med Rehabil.

[18] Thibault-Halman G, Casha S, Singer S, Christie S (2011). Acute management of nutritional demands after spinal cord injury. J Neurotrauma.

[19] Kaufman HH, Rowlands BJ, Stein DK, Kopaniky DR, Gildenberg PL (1985). General metabolism in patients with acute paraplegia and quadriplegia. Neurosurgery.

[20] Kearns PJ, Thompson JD, Werner PC, Pipp TL, Wilmot CB (1992). Nutritional and metabolic response to acute spinal-cord injury. JPEN J Parenter Enteral Nutr.

[21] Devoto G, Gallo F, Marchello C (2006). Prealbumin serum concentrations as a useful tool in the assessment of malnutrition in hospitalized patients. Clin Chem.

[22] Rochester DF, Esau SA (1984). Malnutrition and the respiratory system. Chest.

[23] Lussi C, Frotzler A, Jenny A, Schaefer DJ, Kressig RW, Scheel-Sailer A (2018). Nutritional blood parameters and nutritional risk screening in patients with spinal cord injury and deep pressure ulcer-a retrospective chart analysis. Spinal Cord.

[24] Wilson JR, Grossman RG, Frankowski RF (2012). A clinical prediction model for long-term functional outcome after traumatic spinal cord injury based on acute clinical and imaging factors. J Neurotrauma.

[25] Jones TS, Burlew CC, Johnson JL (2015). Predictors of the necessity for early tracheostomy in patients with acute cervical spinal cord injury: a 15-year experience. Am J Surg.

[26] Rodrigues LF, Moura-Neto V, E Spohr TCLdS. Biomarkers in spinal cord injury: from prognosis to treatment. Mol Neurobiol. 2018;55:6436–48. 10.1007/s12035-017-0858-y 29307082

[27] Hulme CH, Brown SJ, Fuller HR (2017). The developing landscape of diagnostic and prognostic biomarkers for spinal cord injury in cerebrospinal fluid and blood. Spinal Cord.

[28] Badhiwala JH, Ahuja CS, Fehlings MG (2018). Time is spine: a review of translational advances in spinal cord injury. J Neurosurg Spine.

[29] Dionyssiotis Y (2012). Malnutrition in spinal cord injury: more than nutritional deficiency. J Clin Med Res.

[30] Rowan C, Kazemi A (2020). An observational study of feeding practice in ventilated patients with spinal cord injury. Clin Nutr ESPEN.

[31] Eckart A, Struja T, Kutz A (2020). Relationship of nutritional status, inflammation, and serum albumin levels during acute illness: a prospective study. Am J Med.

[32] McMillan DC, Maguire D, Talwar D (2019). Relationship between nutritional status and the systemic inflammatory response: micronutrients. Proc Nutr Soc.

[33] Kwon BK, Streijger F, Fallah N (2017). Cerebrospinal fluid biomarkers to stratify injury severity and predict outcome in human traumatic spinal cord injury. J Neurotrauma.

[34] Yang L, Blumbergs PC, Jones NR, Manavis J, Sarvestani GT, Ghabriel MN (2004). Early expression and cellular localization of proinflammatory cytokines interleukin-1beta, interleukin-6, and tumor necrosis factor-alpha in human traumatic spinal cord injury. Spine (Phila Pa 1976).

[35] Balta S, Demirkol S, Kucuk U (2013). The platelet lymphocyte ratio may be useful inflammatory indicator in clinical practice. Hemodial Int Symp Home Hemodial.

[36] Akkaya E, Gul M, Ugur M (2014). Platelet to lymphocyte ratio: a simple and valuable prognostic marker for acute coronary syndrome. Int J Cardiol.

[37] Altintas O, Altintas MO, Tasal A, Kucukdagli OT, Asil T (2016). The relationship of platelet-to-lymphocyte ratio with clinical outcome and final infarct core in acute ischemic stroke patients who have undergone endovascular therapy. Neurol Res.

[38] Sun T, Shen H, Guo Q (2020). Association between neutrophil percentage-to-albumin ratio and all-cause mortality in critically Ill patients with coronary artery disease. Biomed Res Int.

[39] Cui H, Ding X, Li W, Chen H, Li H (2019). The neutrophil percentage to albumin ratio as a new predictor of in-hospital mortality in patients with ST-segment elevation myocardial infarction. Med Sci Monit Int Med J Exp Clin Res.

[40] Ayling OGS, Ibrahim GM, Alotaibi NM, Gooderham PA, Macdonald RL (2018). Anemia after aneurysmal subarachnoid hemorrhage is associated with poor outcome and death. Stroke.

[41] Warren PM, Campanaro C, Jacono FJ, Alilain WJ (2018). Mid-cervical spinal cord contusion causes robust deficits in respiratory parameters and pattern variability. Exp Neurol.

[42] Young W (2002). Spinal cord contusion models. Prog Brain Res.

[43] Brown R, DiMarco AF, Hoit JD, Garshick E. Respiratory dysfunction and management in spinal cord injury. Respir Care 2006;51:853–68;discussion 69–70. PMC2495152 16867197

