# Supplementary material for: A Nomogram for Predicting Acute Respiratory Failure After Cervical Traumatic Spinal Cord Injury Based on Admission Clinical Findings
Source: Neurocrit Care. 2021 Aug 3;36(2):421–33. doi: 10.1007/s12028-021-01302-4 (PMC8964578; doi:10.1007/s12028-021-01302-4)
Supplement: Supplementary file 1 — (DOCX 16 kb) [file 12028_2021_1302_MOESM1_ESM.docx]

**Table S1. The tolerance and VIF for significant predictors.**

| Predictors | Collinearity statistics | |
| --- | --- | --- |
|  | Tolerance | VIF |
| Level of TSCI | 0.977 | 1.023 |
| AIS | 0.788 | 1.270 |
| Hb | 0.880 | 1.137 |
| PLR | 0.841 | 1.189 |
| NPAR | 0.681 | 1.468 |

Abbreviations: ARF= acute respiratory failure; TSCI= traumatic spinal cord injury; AIS= American Spinal Injury Association Impairment Scale; Hb=Hemoglobin; PLR= platelet to lymphocyte ratio; NPAR= neutrophil percentage to albumin ratio

**Table S2. Comparison of ROC curves**

| Model versus level of TSCI+AIS | |
| --- | --- |
| Difference between areas | 0.112 |
| Standard Error | 0.0448 |
| 95% CI | 0.0246 to 0.200 |
| z statistic | 2.510 |
| Significance level | *p* = 0.0121 |

Pairwise comparison of ROC curves (DeLong’s test)

Abbreviations: ROC= receiver operating characteristic curve; CI= confidence interval
