# Supplementary material for: A Nomogram for Predicting Acute Respiratory Failure After Cervical Traumatic Spinal Cord Injury Based on Admission Clinical Findings
Source: Neurocrit Care. 2021 Aug 3;36(2):421–33. doi: 10.1007/s12028-021-01302-4 (PMC8964578; doi:10.1007/s12028-021-01302-4)
Supplement: Supplementary file 2 — (DOCX 20 kb) [file 12028_2021_1302_MOESM2_ESM.docx]

**Table S3. Baseline characteristics of patients with cervical TSCI in test cohort**

| **Variable** | **Total** | **Without ARF** | **With ARF** |
| --- | --- | --- | --- |
| **Age(years)** † | 57.11±13.12 | 55.43±12.99 | 65.89±10.52 |
| **Sex** ‡ |  |  |  |
| Male | 40(71.4) | 32(68.1) | 8(88.9) |
| Female | 16(28.6) | 15(31.9) | 1(11.9) |
| **Cause of TSCI** ‡ |  |  |  |
| Fall | 27(48.2) | 23(48.9) | 4(44.4) |
| Traffic accidents | 24(42.9) | 19(40.4) | 5(55.6) |
| Other causes | 5(8.9) | 5(10.6) | 0 |
| **Hypertension** ‡ | 11(19.6) | 8(17.0) | 3(33.3) |
| CHD | 2(3.6) | 1(2.1) | 1(11.1) |
| Diabetes | 4(7.1) | 4(8.5) | 0 |
| Smoker | 7(12.5) | 4(8.5) | 3(33.3) |
| **Hospital characteristics** | |  |  |
| SBP(mmHg) † | 132.21±20.44 | 134.94±19.39 | 118.00±20.96 |
| DBP(mmHg) § | 82.00(70.25,87.00) | 83.00(76.00,87.00) | 67.00(62.00,84.00) |
| Heart rate (beats/minute) § | 74.00(70.00,79.50) | 75.00(70.00,80.00) | 72.00(65.00,77.50) |
| **Level of SCI** ‡ |  |  |  |
| C4-C1 | 15(26.8) | 10(21.3) | 5(55.6) |
| C4-C7 | 41(73.2) | 37(78.7) | 4(44.4) |
| **Type of SCI** ‡ |  |  |  |
| Complete | 3(5.4) | 0 | 3(33.3) |
| Incomplete | 53(94.6) | 47(100) | 6(66.7) |
| **AIS** ‡ |  |  |  |
| A or B | 8(14.3) | 3(6.4) | 5(55.6) |
| C or D | 48(85.7) | 44(93.6) | 4(44.4) |
| **Treatment** ‡ |  |  |  |
| Anterior surgery | 7(12.5) | 6(12.8) | 1(11.1) |
| Posterior surgery | 16(28.6) | 15(31.9) | 1(11.1) |
| Nonoperative | 33(58.9) | 26(55.3) | 7(77.8) |
| **Laboratory indices** | |  |  |
| WBC(10^9^/L) § | 9.21(6.83,11.23) | 9.41(6.75,10.70) | 9.00(7.73,12.71) |
| NEU(10^9^/L) § | 7.48(5.46,9.36) | 7.10(5.20,9.24) | 8.60(7.02,10.82) |
| LYM(10^9^/L) § | 1.20(0.88,1.70) | 1.30(1.00,1.72) | 0.40(0.30,1.12) |
| Mon(10^9^/L) § | 0.40(0.20,0.50) | 0.40(0.20,0.50) | 0.33(0.10,0.57) |
| PLT(10^9^/L) § | 205.00(159.00,250.75) | 231.00(166.00,254.00) | 158.00(139.50,183.50) |
| Scr (μmol/L) § | 62.60(48.88,73.35) | 60.90(47.70,72.80) | 71.60(60.10,102.05) |
| Hb(g/L) § | 136.00(123.50,146.00) | 138.00(130.00,147.00) | 123.00(106.50,129.50) |
| RDW-SD(fl) § | 40.15(39.05,41.88) | 40.10(38.90,41.10) | 42.10(40.15,43.80) |
| Albumin(g/dL) † | 3.89±0.39 | 3.96±0.37 | 3.55±0.37 |
| NPAR§ | 20.65(18.35,23.39) | 20.17(16.90,21.90) | 25.60(23.84,26.83) |
| NLR§ | 6.83(3.62,10.93) | 5.64(3.21,9.44) | 16.25(8.72,27.17) |
| SIRI§ | 2.14(0.84,4.31) | 1.96(0.78,4.20) | 3.46(1.77,7.42) |
| PLR§ | 176.58(119.61,271.95) | 174.29(117.06,253.23) | 347.50(118.93,546.67) |

Abbreviations: ARF= acute respiratory failure; TSCI= traumatic spinal cord injury; CHD= chronic heart disease; SBP= systolic blood pressure; DBP= diastolic blood pressure; HR= heart rate; AIS= American Spinal Injury Association Impairment Scale; WBC= white blood cell; NC= neutrophil count; LC= lymphocyte count; MC= monocyte count; Scr= serum creatinine; Hb= hemoglobin; BMI= Body Mass Index; RDW= red cell distribution width; NLR= neutrophil to lymphocyte ratio; PLR= platelet to lymphocyte ratio; NPAR= neutrophil percentage to albumin ratio; SIRI= systemic inflammation response index

†Mean ± SD; ‡ percentage(%); § median(25th, 75th)
